# Effect of nutrition‐based prehabilitation on the postoperative outcomes of patients with esophagogastric cancer undergoing surgery: A systematic review and meta‐analysis

**DOI:** 10.1002/cam4.70023

**Published:** 2024-07-13

**Authors:** Yi Shen, Zhuangzhuang Cong, Qiyue Ge, Hairong Huang, Wei Wei, Changyong Wang, Zhisheng Jiang, Yuheng Wu

**Affiliations:** ^1^ Department of Cardiothoracic Surgery, Jinling Hospital, School of Medicine Southeast University Nanjing China; ^2^ Department of Cardiothoracic Surgery, Jinling Hospital, Affiliated Hospital of Medical School Nanjing University Nanjing China; ^3^ Department of Cardiothoracic Surgery Jinling Hospital Nanjing China

**Keywords:** enhanced recovery after surgery, esophagogastric cancer, meta‐analysis, prehabilitation, preoperative exercise, preoperative nutrition

## Abstract

**Background:**

Meta‐analyses have primarily focused on the effects of exercise‐based prehabilitation on postoperative outcomes and ignored the role of nutritional intervention. In this study, we filled this gap by investigating the effect of nutrition‐based prehabilitation on the postoperative outcomes of patients who underwent esophagectomy and gastrectomy.

**Methods:**

Five electronic databases, namely, PubMed, the Web of Science, Embase, Cochrane Library, and CINAHL, were searched. Adults diagnosed with esophagogastric cancer who were scheduled to undergo surgery and had undergone uni‐ or multimodal prehabilitation, with at least a week of mandatory nutritional intervention, were included. Forest plots were used to extract and visualize the data from the included studies. The occurrence of any postoperative complication was considered the primary endpoint.

**Results:**

Eight studies met the eligibility criteria, with five randomized controlled trials (RCTs) and three cohort studies. In total, 661 patients were included. Any prehabilitation, that is, unimodal (only nutrition) and multimodal prehabilitation, collectively decreased the risk of any postoperative complication by 23% (95% confidence interval [CI] = 0.66–0.90). A similar effect was exclusively observed for multimodal prehabilitation (risk ratio [RR] = 0.78, 95% CI = 0.66–0.93); however, it was not significant for unimodal prehabilitation. Any prehabilitation significantly decreased the length of hospital stay (LOS) (weighted mean difference = −0.77, 95% CI = −1.46 to −0.09).

**Conclusions:**

Nutrition‐based prehabilitation, particularly multimodal prehabilitation, confers protective effects against postoperative complications after esophagectomy and gastrectomy. Our findings suggest that prehabilitation slightly decreases LOS; however, the finding is not clinically significant. Therefore, additional rigorous RCTs are warranted for further substantiation.

## 1 INTRODUCTION

In 2020, 604,100 and 1,089,103 new cases of esophageal cancer (EC) and gastric cancer (GC), respectively, were reported worldwide; this accounts for 3.1% and 5.6% of all cancers. Furthermore, 1,312,869 patients (13.2%) with these two upper gastrointestinal tract (UGI) tumors died in 2020.^1^ Gastrointestinal (GI) tumors exhibit the unique characteristic of being tumors in the primary system of food intake and digestion. These conditions and iatrogenic intervention can exert several adverse effects on the nutrition absorption of patients. Esophagogastric resection is not only a curative‐intent therapy, but also the mainstay of treatment.^2^ However, it is frequently accompanied by poor postoperative outcomes such as pulmonary complications, anastomotic leak, longer length of hospital stay (LOS), and increased mortality rate.^3^, ^4^

To optimize the perioperative procedure, enhanced recovery after surgery (ERAS), a broadly accepted perioperative multidisciplinary care procedure, has been developed and proven to help accomplish earlier hospital discharge without hampering postoperative care for esophagectomy and gastrectomy.^5^, ^6^ Although, data are inadequate to draw solid conclusions, the ERAS society has still given a moderate recommendation grade for prehabilitation for esophagectomy and major abdominal surgery.^7^ Surgical prehabilitation occurs between the time of cancer diagnosis and the start of surgical treatment; it includes nutritional support such as oral nutritional supplement (ONS) and diet counseling, exercise support such as aerobic and resistance exercise, and psychological support such as anxiety attenuation to optimize the preoperative functional capacity.^8^, ^9^ In patients receiving GI surgery, the risk of malnutrition is present both pre and postoperatively.^10^ Perioperative malnutrition frequently indicates higher LOS postoperatively, morbidity, mortality, and medical costs.^11^, ^12^ This makes nutritional prehabilitation a vital measure to prepare or optimize patients for surgery, and not necessarily to replace nutritional deficits.^13^

Most meta‐analyses^14^, ^15^, ^16^, ^17^ have only focused on studies on exercise prehabilitation, with the effects on short‐ and long‐term postoperative outcomes being inconsistent. In one meta‐analysis,^18^ researchers investigated the effect of multimodal prehabilitation on the postoperative outcomes of patients who underwent hepatobiliary, colorectal, and UGI cancer surgery; however, they only included three studies on UGI cancer, thereby providing less persuading evidence. Nevertheless, in a systematic review and meta‐analysis, Gillis et al.^19^ have reported promising results: after colorectal surgery, only nutritional prehabilitation or combination with an exercise program decreased hospital stay by 2 days.

Therefore, in the present systematic review and meta‐analysis, we investigated the effects of unimodal or multimodal prehabilitation, with mandatory nutrition prehabilitation, on the clinical outcomes of patients with esophagogastric cancer who were awaiting surgery. The primary objective was to observe the changes in postoperative complications. The secondary objective was to assess the changes in LOS, readmission, and mortality.

## 2 METHODS

The reporting guidance of The Preferred Reporting Items for Systematic Reviews and Meta‐Analyses was followed for this systematic review and meta‐analysis.^20^ The review protocol was recorded and registered in PROSPERO (registration number: CRD42022314766).

### 2.1 Objective

The primary objective of this meta‐analysis was to investigate the effect of nutritional prehabilitation with or without exercise and/or psychological support on the postoperative complications of patients with gastroesophageal cancer undergoing surgery. Our second objective was to determine whether prehabilitation can decrease LOS, readmission, and mortality compared with patients who received conventional care.

### 2.2 Search strategy

Five electronic databases, namely, PubMed, the Web of Science, Embase, Cochrane Library, and CINAHL, were searched. Without restrictions on countries or study types, all English publications until June 1, 2023, were searched. Furthermore, the references of all the selected studies and associated reviews were independently screened to identify additional studies that were omitted in the original search. The search strategy was established based on “P” (patients with EC and/or GC) and “I” (prehabilitation, i.e., preoperative nutrition with or without exercise and/or psychological support) in the PICOS principle (Appendix S1).

### 2.3 Study selection

After performing the initial search and removing duplicates, two reviewers (YHW and QYG) independently assessed the titles and abstracts of full‐text reviews. All disagreements were resolved by a third author (ZZC). All eligible studies, including references from the selected studies and reviews, were included. Adults (more than 18 years of age) with esophagogastric cancer who were planning to undergo surgery were included. Studies with multiple cancer types that were not separately analyzed were excluded. Nutritional prehabilitation, defined as the preoperative application of ONS or enteral nutrition (EN) with or without dietary advice for at least 7 days, thereby altering macronutrient (carbohydrate, protein, and fat) intake, was mandatory. Exercise prehabilitation included preoperative aerobic exercises, strength or resistance exercises, and inspiratory muscle training at the hospital or home. Psychological prehabilitation included preoperative consultation, motivational interviewing, and psychometric screening. Only studies that included nutritional prehabilitation were considered. Unimodal prehabilitation only included nutrition, whereas, multimodal included two or three interventions, with nutrition being one intervention. In terms of nutritional prehabilitation, the patients in the control group were not subjected to any intervention (routine daily diet as a negative control) or subjected to only nutritional counseling. However, some high‐risk patients (<50% in the control group) were allowed to undergo the same interventions performed in the prehabilitation group. Preoperative exercise and psychological interventions were both negative controls in the control group. Furthermore, postoperative patient management was similar in both groups, for example, both underwent the ERAS program. Only original randomized controlled trials (RCTs) and cohort studies were included.

### 2.4 Data extraction

The data were independently extracted by two reviewers (YHW and QYG). Any disagreements were resolved by a third reviewer (ZZC). The data extraction sheet was assessed on two studies that were randomly selected. If available, the following data were extracted: (1) baseline characteristics, (2) intervention characteristics, and (3) reported outcomes. The corresponding author was contacted to address missing data. Data management and extraction were performed using Zotero 6.0.26 (Corporation for Digital Scholarship, USA) and Excel version 2305 (Microsoft, USA).

### 2.5 Quality assessment

The risk of bias in RCTs was assessed using the Cochrane Collaboration's tool, with six domains. Bias was graded as high, low, or unclear risk.^21^ The assessment was completed using Review Manager (RevMan) 5.4.1 (Cochrane, UK). The Newcastle–Ottawa Scale (NOS) was used to assess the cohort studies. A star system (a maximum of 9 stars) was used to judge the detection of selection, comparability, and exposure or outcome.^22^ Higher stars indicate a lower risk of bias. Two reviewers (YHW and QYG) conducted the assessment. Any disagreements were resolved by a third reviewer (ZZC).

### 2.6 Statistical analysis

Forest plots were generated to investigate the effects of prehabilitation on postoperative outcomes. Pooled risk ratio (RR) was used for categorical data, standard mean difference for varying units, and weighted mean difference (WMD) for continuous variables, with a 95% confidence interval (CI). The *I* ^2^ test was used to assess heterogeneity. If *I* ^2^ < 50% or *p* > 0.1, a fixed‐effects model was applied, otherwise, a random‐effects model was applied. Subgroup analysis was stratified by intervention, cancer type, surgical care, and research design. If specific studies only provided median and interquartile range, the mean and standard deviation (SD) were estimated.^23^, ^24^ If more than 10 studies were included, the Egger test was used to assess publication bias.^25^ RevMan 5.4.1 (Cochrane, UK) was used to perform data analysis. A *p* < 0.05 indicated statistical significance.

## 3 RESULTS

### 3.1 Search outcomes

**FIGURE 1:**
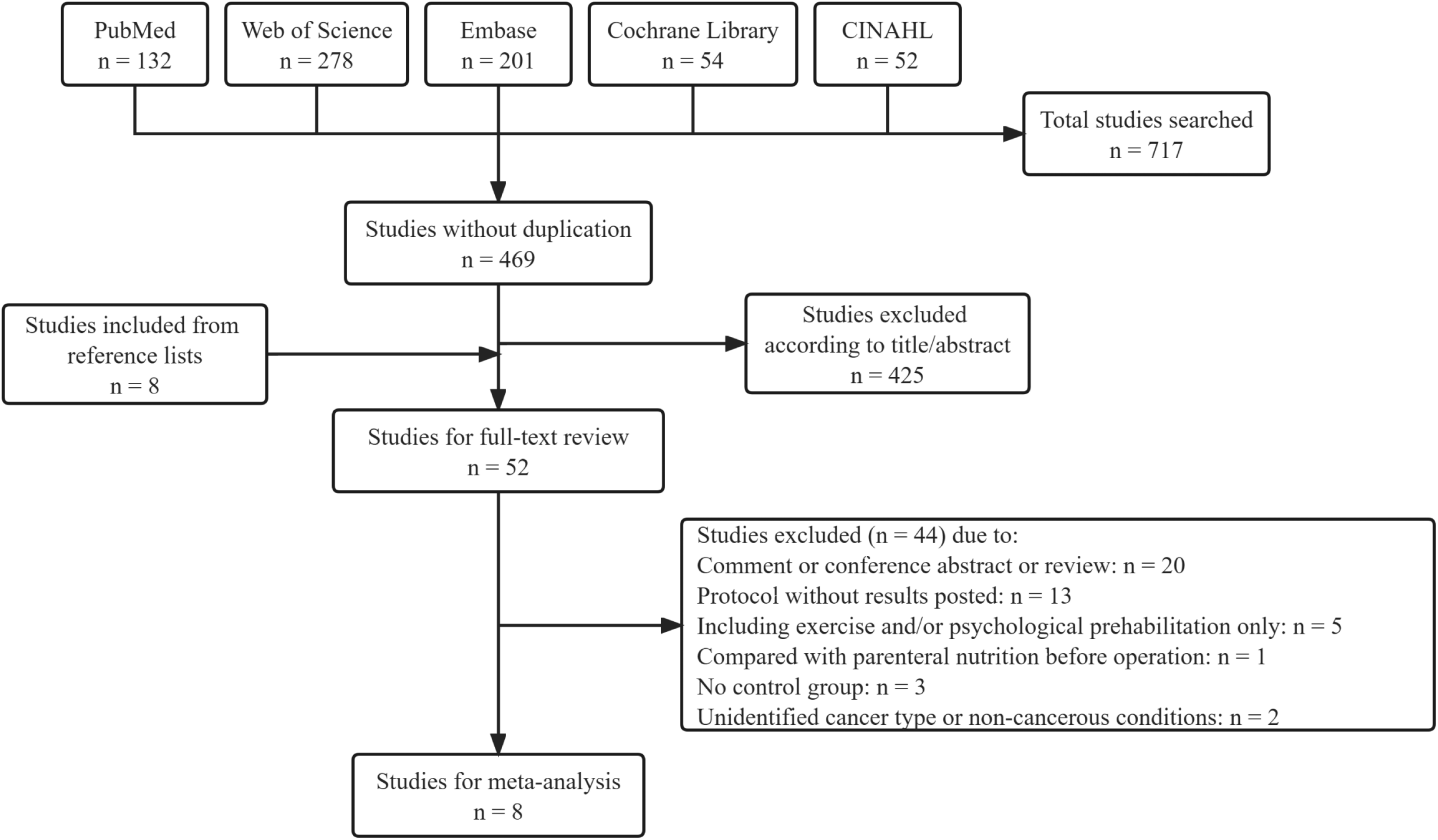
Preferred Reporting Items for Systematic Reviews and Meta‐Analyses flow diagram of study selection.

After screening the five online databases based on the pre‐developed search strategy, 717 studies were identified: 132 in PubMed, 278 in the Web of Science, 201 in Embase, 54 in Cochrane Library, and 52 in CINAHL. After removing duplicate studies and studies whose titles or abstracts did not meet the inclusion criteria and adding eight possibly qualified studies from the references, 52 studies were included for full‐text review. In total, eight studies^26^, ^27^, ^28^, ^29^, ^30^, ^31^, ^32^, ^33^ (five RCTs and three cohort studies) met the inclusion criteria and were included in the final analysis via additional screening (Figure 1).

### 3.2 Baseline characteristics

Eight studies from five countries between 2015 and 2022 were analyzed (Table 1). Among them, five were RCTs^27^, ^28^, ^29^, ^30^, ^32^ and three were cohort studies.^26^, ^31^, ^33^ Halliday et al.^33^ used propensity scores to match the prehabilitation and control groups; however, no significant differences were observed in the demographic factors, except for respiratory comorbidity before matching. Therefore, we adopted the data of the unmatched groups in the subsequent analysis. In total, 661 operative patients were included, with 351 in the prehabilitation group and 310 in the control group. The mean or median age range was 60.5–68, except for the study conducted by Wang et al.^30^ who did not calculate the mean or median age. More than 50% of the patients were diagnosed with esophageal and esophagogastric junction cancer (58%, 385/661); in contrast, the remaining patients were diagnosed with GC (42%, 276/661). Furthermore, 56%–100% of patients in the selected studies received at least one type of neoadjuvant therapy; however, two studies^29^, ^30^ did not provide any information on neoadjuvant therapy. Five studies^26^, ^27^, ^29^, ^32^, ^33^ were conducted under the ERAS setting, comprising immediate feeding, early mobilization, and early drain and tube removal postoperatively,^34^ in the intervention and control groups.

**TABLE 1:** Baseline characteristics.

Study and location	Study design	Study groups	Surgical care	Sample size	Age (years)	Men, *n* (%)	Cancer type, *n* (%)	Neoadjuvant therapy type, *n* (%)	Surgery type, *n* (%)	Nutrition screening and assessment	Preoperative malnutrition condition, *n* (%)	Preoperative physical condition
F. He, 2022, China	Single‐blind RCT, single center	Unimodal; I: 1 week preop nutrition support; C: dietary advice	ERAS	I: 31; C: 35	Mean (SD) = 63.2 (12.0) in I, 60.5 (9.4) in C	24 (77.4) in I, 23 (65.7) in C	Gastric cancer in I (100) and C (100)	NA	Gastrectomy in I (100) and C (100)	NRS2002	NA	NA
L. J. Halliday, 2021, UK	Propensity score matched cohort (I: prospective, C: retrospective and prospective), single center	Multimodal; I: >1 week preop exercise, nutrition and psychological support (PREPARE programme); C: ERAS	ERAS	Unmatched: I: 72; C: 39; matched: I: 38; C: 38	Unmatched: median (IQR) = 68 (61–73) in I, 67 (62–74) in C; matched: median (IQR) = 69 (60–73) in I, 68 (61–74) in C	NA	Esophageal and esophagogastric junction cancer in I (100) and C (100)	Unmatched: NAC 63 (88) in I, 21 (87) in C; matched: NAC 33 (87) in I, 33 (87) in C	Curative surgery for resectable esophageal cancer in I (100) and C (100)	An assessment of nutritional status including identification and stratification of nutritional risk	NA	NA
T. Janssen, 2021, Netherlands	Cohort (I: prospective, C: retrospective), single center	Multimodal; I: 10 weeks preop exercise, nutrition and psychological support (PREPARE programme); C: optimized ERAS	Optimized ERAS^a^	I: 52; C: 43	Median (IQR) = 64 (NA) in I, 65 (NA) in C	39 (75.0) in I, 35 (81.4) in C	Esophageal cancer in I (100) and C (100)	NAC 3 (5.8) in I, 0 (0) in C; NAR 0 (0) in I, 1 (2.3) in C; NCRT 49 (94.2) in I, 42 (97.7) in C	MIE‐IL in I (100) and C (100)	NS^b^	NA	*V*O_2max_ (mL/kg/min), mean = 25.79 in I, NA in C; MIP (% of predicted), mean = 108 in I, NA in C
K. Liu, 2020, China	Pilot single‐blind RCT, single center	Unimodal; I: 1 week preop nutrition support for all patients+30d postdischarge HEN; C: 1 week preop nutrition support for patients with NRS2002 score ≥3	ERAS	I: 26; C: 24	Mean (SD) = 62.04 (5.12) in I, 64.58 (5.87) in C	21 (80.8) in I, 14 (58.3) in C	Esophageal cancer in I (100) and C (100)	NAT 13 (50) in I, 15 (62.5) in C	McKeown 19 (73.1) in I, 13 (54.2) in C; Ivor‐Lewis 7 (26.9) in I, 11 (45.8) in C	BMI; albumin; NRS2002	0 (0) in I, 1 (4.2) in C according to BMI <18.5 kg/m^2^	NA
L. C. Dewberry, 2019, USA	Retrospective cohort, single center	Multimodal; I: 10–18 weeks preop exercise, nutrition and psychological support (STRENGTH programme); C: standard care	Traditional care	I: 11, C: 11	Mean (SD) = 67.3 (NA) in I, 62.7 (NA) in C	9 (81.8) in I, 9 (81.8) in C	Esophageal cancer in I (100) and C (100)	NCRT in I (100) and C (100)	Ivor‐Lewis 9 (81.8) in I, 10 (90.9) in C; transhiatal 1 (9.1) in I, 0 (0) in C; three‐hole 1 (9.1) in I, 0 (0) in C; others 0 (0) in I, 1 (9.1) in C	ASPEN malnutrition assessment tool	NA	Measured by BDI survey and grip strength assessment but no measurements were reported
E. M. Minnella, 2018, Canada	Pragmatic single‐blind RCT, single center	Multimodal; I: 3–4 weeks (depending on whether to receive NAC) preop exercise, nutrition and psychological support; C: ERAS	ERAS	I: 26, C: 25	Mean (SD) = 67.3 (7.4) in I, 68.0 (11.6) in C	18 (69) in I, 20 (80) in C	Esophageal cancer 20 (77) in I, 21 (84) in C; gastric cancer 6 (23) in I, 4 (16) in C	NAT 20 (77) in I, 15 (60) in C	Esophagectomy 18 (75) in I, 21 (84) in C; partial gastrectomy 4 (17) in I, 2 (8) in C; total gastrectomy 2 (8) in I, 2 (8) in C^c^	Three‐day food record; PG‐SGA; lean body mass and fat; total energy expenditure	NA	6MWD (m), mean (SD) = 452.1 (83.4) in I; 449.2 (83.9) in C
Q. Zhao, 2018, China	RCT, single center	Unimodal; I: 1 week preop nutrition support; C: 1 week preop routine diet	Traditional care	I: 33, C: 33	Mean (SD) =62 (NA) and ranging from 26 to 74 in all participants	57 males and 9 females in all participants	AEG in I (100) and C (100)	NCRT in I (100) and C (100)	NA	NRS2002; PG‐SGA	18 (54.5) in I, 19 (57.6) in C according to the NRS2002 score	NA
F. Wang, 2015, China	RCT, single center	Unimodal; I: 1 week preop nutrition support, C: standard care	Traditional care	I: 100, C: 100	38–72 in all participants	130 males and 70 females in all participants	Gastric cancer in I (100) and C (100)	NA	NA	NA	NA	NA

Abbreviations: 6MWD, 6‐minute walk distance; AEG, adenocarcinomas of esophagogastric junction; ASPEN, American Society for Parenteral and Enteral Nutrition; BDI, Beck Depression Inventory; BMI, body mass index; C: control; ERAS, enhanced recovery after surgery; HEN, home enteral nutrition; I, intervention; IQR, interquartile range; MIE‐IL, minimally invasive Ivor‐Lewis esophagectomy; MIP, maximum inspiratory pressure; NA, not available; NAC, neoadjuvant chemotherapy; NAR: neoadjuvant radiotherapy; NAT, neoadjuvant therapy; NCRT, neoadjuvant chemoradiotherapy; NRS2002, nutrition risk screening 2002; NS, not specific; PG‐SGA, Patient‐Generated Subjective Global Assessment; preop, preoperative; RCT, randomized controlled trial; SD, standard deviation.

^a^ Starting oral feeding on postoperative day one after a minimally invasive Ivor‐Lewis esophagectomy.

^b^ This study followed PREPARE prehabilitation programme, firstly introduced at St Mary's Hospital, London (Halliday,^33^). When certain details were not specifically described, they were considered to be the same as Halliday et al.^33^

^c^ Missing data for two patients (both in the prehabilitation group) who did not have surgery.

### 3.3 Intervention characteristics

As demonstrated in Tables 1 and 2, all interventions in the included studies lasted for at least 1 week, with the longest one lasting for 10–18 weeks.^31^ The actual prehabilitation duration was assessed in three studies,^27^, ^29^, ^31^ with a mean or median time range of 7.6–63 days. In four studies,^26^, ^27^, ^31^, ^33^ multimodal prehabilitation was implemented, with all studies including preoperative exercise, nutritional support, and psychological support. Halliday et al.^33^ and Janssen et al.^26^ adopted the same program, that is, the PREPARE program, whereas Dewberry et al.^31^ adopted the STRENGTH program. Four RCTs from China^28^, ^29^, ^30^, ^32^ employed unimodal prehabilitation that is, nutrition‐only prehabilitation. In contrast, Liu et al.^32^ additionally administered 30‐day post‐discharge home EN to the patients in the prehabilitation group. Except for one study,^31^ which did not explicitly report the type of nutrition prehabilitation, ONS was the only choice of nutrition in five studies.^27^, ^28^, ^29^, ^30^, ^32^ Furthermore, in addition to ONS, the PREPARE program^26^, ^33^ also allowed EN via jejunostomy. The exercise intervention primarily comprised a mixture of home‐based, personalized aerobic, and strength or resistance exercises preoperatively^26^, ^27^, ^33^; however, the Be Fit/Be Well exercise program was not well‐defined.^31^ The preoperative psychological intervention primarily included psychometric screening, consultation, and motivational interviewing.^26^, ^27^, ^31^, ^33^ In seven studies, a negative control group was included.^26^, ^27^, ^28^, ^29^, ^30^, ^31^, ^33^ However, Liu et al.^32^ administered the same preoperative nutritional support as the trial group to patients in the control group, with a Nutrition Risk Screening 2002 (NRS2002) score of ≥3.

**TABLE 2:** Intervention characteristics.

Study and location	Type of nutritional supplement	Nutrition prescription	Preoperative supplemental energy (kcal) /protein (g) intake	Exercise type	Exercise prescription	Goal of exercise	Psychological support	Length of prehabilitation (d)	Monitoring of intervention	Compliance with prehabilitation	Control group	Reported outcomes
F. He, 2022, China	ONS (TPFD, Ruidai)	Before surgery, ONS 500 mL/day (one bag)	Energy 450 kcal; protein 17 g (one ONS pack)	NA	NA	NA	NA	Mean (SD) = 7.6 (NA)	Nutrition: the amount of daily ONS intake was recorded and checked by the dietitian during the consultation sessions	NA	Before surgery, dietary advice	Primary outcome: the incidence of feeding intolerance; others: the rate of energy supply by enteral nutrition up to 50% of the target daily energy requirement (25 kcal/kg/day) within 5 days after surgery, postoperative gastrointestinal symptom rate, postoperative laboratory measurements, postoperative complication rate, readmission rate at 1 month
L. J. Halliday, 2021, UK	ONS or EN via a jejunostomy	Before surgery, a plan was agreed based on symptoms, dietary eating habits, and nutritional deficiencies	NA	Aerobic and strength/resistance training	Before surgery, home‐based, personalized; patients received training on how to undertake the exercises and how to selfregulate the intensity using the Borg scale rating of perceived exertion, with a target range of 13–15	A minimum of 600 MET min/week, with the aim of increasing to 1200 MET min/week	Before surgery, psychometric screening; consultation; motivational interviewing techniques	NA	Exercise: weekly telephone touch‐point from an exercise therapist; nutrition: weekly or fortnightly phone calls from the dietitian	Exercise: mean (SD) = 55% (29.8) during NAC and 66% (35.9) after it was completed^a^	Before surgery, no intervention	Primary outcome: 60‐day postoperative complications; others: 60‐day pulmonary complications, severe complications (Clavien‐Dindo grade 3 and higher), length of stay, 30‐day readmission rate
T. Janssen, 2021, Netherlands	NS^b^	Before surgery, nutritional goals were assessed by dieticians using the Harris‐Benedict formula to estimate the total caloric and protein need	NA	NS^b^	NS^b^	Goals were determined for each patient individually and adjusted each week throughout the programme	Before surgery, mental state, wellbeing and social support were discussed	NA	Exercise: patients were contacted by the physiotherapists each week to evaluate a weekly training schedule	NA	Before surgery, no intervention	Functional recovery, length of hospital stay, hospital readmission (within 30 days), ICU readmission (same hospital stay), weight loss, 90‐day mortality, postoperative complications (within 30 days), cardiopulmonary complications, anastomotic leakage
K. Liu, 2020, China	ONS (Peptisorb, nutricia)	Before surgery, ONS 500–1000 mL/day; after discharge, ONS 500 mL/day via oral intake or jejunostomy tube	Non‐protein energy 23–30 kcal/kg/day; protein 1.2–1.5 g/kg/day	NA	NA	NA	NA	NA	Nutrition: nutritionist monitored the adherence and addressed issues by telephone calls after discharge	NA	Before surgery, ONS 500–1000 mL/day if NRS2002 score ≥3	Primary outcome: weight change before and after esophagectomy; others: body mass index, lean body mass, appendicular skeletal muscle mass index, nutrition‐related complications, quality of life
L. C. Dewberry, 2019, USA	NA	During NCRT and before surgery, nutrition consult and nutrition pathway	NA	NA	During NCRT and before surgery, Be Fit/Be Well exercise programme referral	NA	During NCRT and before surgery, psychology referral; distress screening at each infusion session	Median (IQR) = 63 (NA)	Nutrition: the dietician had weekly meetings with patients; psychology: the distress screen was distributed by the receptionists and reviewed by the social workers at each infusion visit; the symptom questionnaire required the clinic nurse to call each patient between completion of neoadjuvant therapy and restaging	Nutrition: all patients were compliant with the nutritional component of the programme; exercise: 63.6% participated in the exercise programme	Before surgery, no intervention	Primary outcome: length of time from neoadjuvant therapy to surgery; others: clinical and pathological response after neoadjuvant therapy, clinical outcomes (nutritional status, type of surgery, length of surgery, estimated blood loss, complications, readmission rates, length of stay, and mortality), 30‐ and 90‐d time points readmission rates and mortality
E. M. Minnella, 2018, Canada	ONS (whey protein supplement (immunocal; immunotecInc), ensure plus, boost, plus, resource fruit beverage, and beneprotein powder as needed)	Before surgery, food‐based dietary advice; samples of dietary supplements as needed; strategies to optimize dietary energy and protein intake according to current standard hospital protocols	Approximately 20% of total energy requirements; protein 1.2–1.5 g/kg IBW/d	Aerobic and strength/resistance training	Before surgery, home‐based, individualized; 4 times/week; aerobic exercise, 30 min (including 5‐min warm‐up and 5‐min cooldown) of moderate continuous training 3 days/week; exercise modalities were brisk walk, jogging, or cycling; strengthening activity, 1 day/week, 30 min (including 5‐min flexibility and 5‐min stretching) of 3 sets of 8–12 repetitions for 8 muscle groups using an elastic band as resistance	Aerobic exercise, 12–13 on rated perceived exertion (range 6–20 on the Borg Rating of Perceived Exertion Scale); strengthening activity, a moderate‐intensity effort resistance level, rated as 5–6 on a 10‐point scale	Before surgery, consultation	Median (IQR) = 36 (17–73)	Nutrition and exercise: participants were provided with a logbook to record all activities; the kinesiologist and the nutritionist monitored the adherence and addressed issues or doubts by weekly telephone calls	63%^c^	Before surgery, no intervention	Primary outcome: change in functional capacity over time; others: postoperative complications at 30 days, length of hospital stay, 30‐day hospital visits, readmission rate, death, full adherence to the planned neoadjuvant chemotherapy, overall compliance with prehabilitation
Q. Zhao, 2018, China	ONS (EN suspension TPF, nutrison fiber)	Before surgery, ONS 500 mL/day+ routine preoperative diet	Energy 25–30 kcal/kg/day, protein 1.0–1.5 g/kg/day	NA	NA	NA	NA	NA	NA	NA	Before surgery, no intervention	Nutritional indicators, intestinal barrier indicators, postoperative recovery
F. Wang, 2015, China	ONS (Nutrison, German Nutricia Export B.V.)	Before surgery, Nutrison taken orally	Energy 1000 kcal/day; protein NA	NA	NA	NA	NA	NA	NA	NA	Before surgery, no intervention	Complications, clinical laboratory measurements, nutritional indicators, immunologic indicators, cytokine levels

Abbreviations: EN, enteral nutrition; IBW, ideal body weight; IQR, interquartile range; MET, metabolic equivalent of task; NA, not available; NAC, neoadjuvant chemotherapy; NCRT: neoadjuvant chemoradiotherapy; NRS2002, nutrition risk screening 2002; NS, not specific; ONS, oral nutritional supplement; SD, standard deviation.

^a^ Caculated as the volume of exercise completed by the patient each week in MET minutes/week divided by the prescribed volume of exercise each week in MET minutes/week.

^b^ This study followed PREPARE prehabilitation programme, firstly introduced at St Mary's Hospital, London (L. J. Halliday, 2021). When certain details were not specifically described, they were considered to be the same as Halliday et al.^33^

^c^ Integrating both exercise (number of weekly training sessions completed) and nutrition (adherence to the prescribed protein supplementation).

### 3.4 Risk of bias

The Cochrane Collaboration's tool was used in five RCTs^27^, ^28^, ^29^, ^30^, ^32^ (Figure S1a,b). Owing to the nature of the intervention, masking participants or healthcare professionals was impossible. As a result, a high risk of performance bias was observed. Wang et al.^30^ and Zhao et al.^28^ did not report concrete baseline statistics, resulting in significant underlying differences in the baseline; therefore, these studies exhibited high risk in terms of other biases. The reporting bias of most RCTs^28^, ^29^, ^30^, ^32^ was unclear because they did not provide the protocols to evaluate the predetermined outcomes, except for one^27^ RCT that mentioned that the trial protocol was at low risk. NOS was used to assess the three cohort studies^26^, ^31^, ^33^ (Figure S1c). One study^26^ lost a star in comparability because control for the pTNM stage between the groups was not completed (*p* = 0.014). Another study^31^ had a retrospective design and the outcomes of interest were present at the start of the study; therefore, it lost a star in selection. Publication bias was not evaluated because only eight studies were included (<10).

### 3.5 Quantitative synthesis of outcomes

#### 3.5.1 Postoperative complications

**FIGURE 2:**
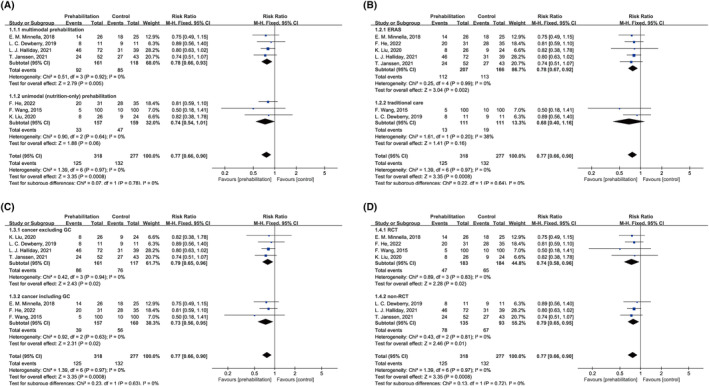
Effects of any prehabilitation on any postoperative complication in patients with esophagogastric cancer after esophagectomy and gastrectomy (A) stratified by multimodal and unimodal (only nutrition) prehabilitation; (B) in an ERAS and traditional care setting; (C) with cancer excluding GC and cancer including GC; and (D) in RCTs and non‐RCTs. CI, confidence interval; ERAS, enhanced recovery after surgery; GC, gastric cancer; RCT, randomized controlled trial.

**FIGURE 3:**
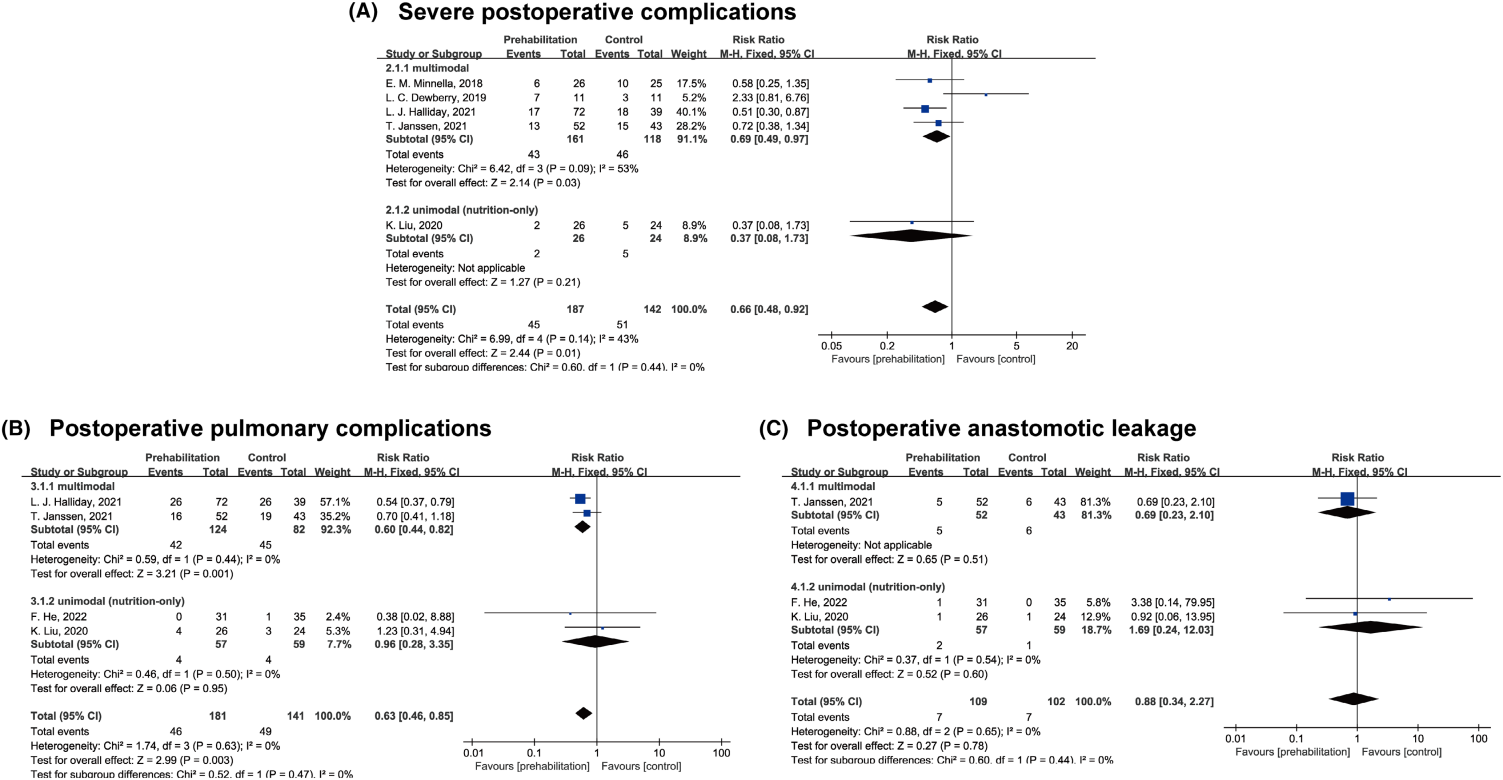
Effect of multimodal and unimodal (only nutrition) prehabilitation on (A) severe postoperative complications; (B) postoperative pulmonary complications; and (C) postoperative anastomotic leakage in patients with esophagogastric cancer after esophagectomy and gastrectomy. CI, confidence interval.

Seven studies^26^, ^27^, ^29^, ^30^, ^31^, ^32^, ^33^ revealed postoperative complications during the hospital stay^29^, ^30^, ^31^, ^32^ or within 30^26^, ^27^ or 60^33^ days postoperatively. All these studies were included in the pooling results (Table 3 and Figures 2 and 3). In almost all studies,^26^, ^27^, ^31^, ^32^, ^33^ postoperative complications were graded using the Clavien–Dindo classification (CDC). In the seven analyzed studies, the rate of any postoperative complication was 39.3% (125/318) and 47.7% (132/277) in the prehabilitation and control groups, respectively.

**TABLE 3:** Analyzed results.

Study and location	Postoperative complications, *n* (%)	Hospital length of stay (LOS, days)	Hospital readmission, *n* (%)	Mortality, *n* (%)
F. He, 2022, China	During hospital stay: any complication^a^ 20 (64.5) in I, 28 (80.0) in C; pulmonary complication 0 (0) in I, 1 (2.9) in C; anastomotic leakage 1 (3.2) in I, 0 (0) in C	NA	30 days: 0 (0) in I, 1 (2.9) in C	NA
L. J. Halliday, 2021, UK	Within 60 days after surgery in unmatched groups: any complication 46 (68) in I, 31 (79) in C; pulmonary complication 26 (36) in I; 26 (67) in C; severe complication^b^ 17 (24) in I, 18 (46) in C; in matched groups: any complication 24 (63) in I, 31 (82) in C; pulmonary complication 12 (32) in I; 26 (68) in C; severe complication^b^ 12 (32) in I, 18 (47) in C	Unmatched: median (IQR) = 10 (8–17) in I, 13 (11–20) in C; matched: median (IQR) = 10 (8–17) in I, 13 (11–20) in C; calculated unmatched: mean (SD) = 11.8 (6.8) in I, 14.8 (6.9) in C; calculated matched: mean (SD) = 11.8 (6.9) in I, 14.8 (6.9) in C	30 days: unmatched: 13 (18) in I, 3 (8) in C; matched: 9 (24) in I, 3 (8) in C	NA
T. Janssen, 2021, Netherlands	Within 30 days after surgery according to CDC:I0 (0) in I, 1 (2.3) in C; II11 (21.2) in I, 11 (25.6) in C; III9 (17.3) in I, 6 (14.0) in C; IV4 (7.7) in I, 8 (18.6) in C; V0 (0) in I, 1 (2.3) in C; any complication 24 (46.2) in I, 27 (62.8) in C; pulmonary complication 16 (30.8) in I, 19 (44.2) in C; anastomotic leakage 5 (9.6) in I, 6 (14.0) in C	Median (IQR) = 7 (6–10) in I, 8 (7–10) in C; calculated mean (SD) = 7.7 (3.0) in I, 8.4 (2.3) in C	30 days: 5 (9.6) in I, 6 (14.3) in C	90 days: 1 (1.9) in I, 1 (2.3) in C
K. Liu, 2020, China	During hospital stay according to CDC:II6 (23.1) in I, 4 (16.7) in C; III1 (3.8) in I, 5 (20.8) in C; IV1 (3.8) in I, 0 (0) in C; any complication 8 (30.8) in I, 9 (37.5) in C; pulmonary complication 4 (15.4) in I, 3 (12.5) in C; anastomotic leakage 1 (3.8) in I, 1 (4.2) in C	Mean (SD) = 13.77 (3.72) in I, 13.33 (3.53) in C	1 (3.8) in I, 0 (0) in C (days are NA)	30d: 0 (0) in I, 0 (0) in C; 90d: 0 (0) in I, 0 (0) in C
L. C. Dewberry, 2019, USA	During hospital stay according to CDC:I0 (0) in I, 2 (18.2) in C; II1 (9.1) in I, 4 (36.4) in C; III4 (36.4) in I, 3 (27.3) in C; IV3 (27.3) in I, 0 (0) in C; any complication 8 (72.7) in I, 9 (81.8) in C	Median (IQR) = 13.0 (NA) in I, 10.0 (NA) in C	30 days: 0 (0) in I, 2 (18.2) in C; 90 days: 2 (18.2) in I, 3 (27.3) in C	30 days: 0 (0) in I, 0 (0) in C; 90 days: 1 (9.1) in I, 1 (9.1) in C
E. M. Minnella, 2018, Canada	Within 30 days after surgery according to CDC:I2 (8) in I, 0 (0) in C; II6 (25) in I, 8 (32) in C; III3 (13) in I, 7 (28) in C; IV3 (12) in I, 1 (4) in C; V0 (0) in I, 2 (8) in C; any complication 14 (58) in I, 18 (72) in C	Median (IQR) = 8.0 (5.75–11.75) in I, 7.0 (5.5–12.5) in C; calculated mean (SD) = 8.5 (4.7) in I, 8.4 (5.5) in C	1 (4) in I, 2 (8) in C (days are NA)	In hospital: 0 (0) in I, 2 (8) in C
Q. Zhao, 2018, China	NA	Median (IQR) = 7 (6–8) in I, 8 (6–10) in C; calculated mean (SD) = 7 (1.5) in I, 8 (3.1) in C	NA	NA
F. Wang, 2015, China	During hospital stay: abdominal distension and pain 5 (5.19) in I, 10 (9.55) in C (no occurrence of acute intestinal obstruction, intestinal fistula, or other complications)	NA	NA	NA

Abbreviations: C, control; CDC, Clavien‐Dindo classification; I, intervention; IQR, interquartile range; NA, not available; SD, standard deviation.

^a^ Including symptoms of feeding intolerance and other non‐feeding related complication.

^b^ CDC III and higher.

Any prehabilitation, that is, unimodal combined with multimodal prehabilitation, significantly decreased the risk of any postoperative complication by 23% (95% CI = 0.66–0.90), with little heterogeneity (*I* ^2^ = 0%, *p* = 0.97; Figure 2). This effect was consistent and not affected by the study design. Both RCTs and non‐RCTs suggested that prehabilitation can effectively mitigate any postoperative complication (Figure 2D). Furthermore, the outcomes where only multimodal prehabilitation studies were analyzed were similar to the overall pooling result (RR = 0.78, 95% CI = 0.66–0.93; Figure 2A). However, the results were not statistically significant (RR = 0.74, 95% CI = 0.54–1.01; Figure 2A) in studies on unimodal (only nutrition) prehabilitation. Stratification by whether the ERAS program was applied in perioperative patient care revealed a significant decrease in any postoperative complication favoring prehabilitation (RR = 0.78, 95% CI = 0.67–0.92) under the ERAS settings (Figure 2B). Prehabilitation significantly decreased the risk of any postoperative complication in patients with EC and GC (Figure 2C).

Severe postoperative complications were defined as CDC grade III or higher and were observed in five studies.^26^, ^27^, ^31^, ^32^, ^33^ Figure 3A illustrates a significant decrease in severe postoperative complications favoring any prehabilitation (RR = 0.66, 95% CI = 0.48–0.92) and multimodal prehabilitation (RR = 0.69, 95% CI = 0.49–0.97). Figure 3B illustrates a decrease in postoperative pulmonary complications^26^, ^29^, ^32^, ^33^ in any prehabilitation (RR = 0.63, 95% CI = 0.46–0.85) and multimodal prehabilitation (RR = 0.60, 95% CI = 0.44–0.82). In three studies^26^, ^29^, ^32^ (*n* = 211) in which anastomotic leakage was assessed, the risk of postoperative anastomotic leakage in any prehabilitation group decreased by 12%; however, the result was not statistically significant (95% CI = 0.34–2.27; Figure 3C).

#### 3.5.2 LOS

**FIGURE 4:**
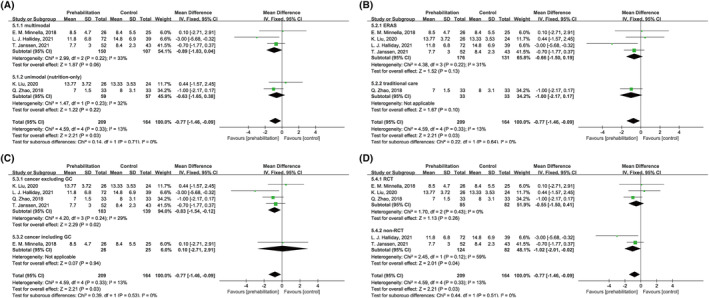
Effect of any prehabilitation on LOS in patients with esophagogastric cancer after esophagectomy and gastrectomy (A) stratified by multimodal and unimodal (only nutrition) prehabilitation; (B) in an ERAS and traditional care setting; (C) with cancer excluding GC and cancer including GC; and (D) in RCTs and non‐RCTs. LOS, length of hospital stay; CI, confidence interval; ERAS, enhanced recovery after surgery; GC, gastric cancer; RCT, randomized controlled trial.

LOS was reported in six studies^26^, ^27^, ^28^, ^31^, ^32^, ^33^ (Table 3 and Figure 4). However, SD was not reported in one study.^31^ Therefore, the results of five studies^26^, ^27^, ^28^, ^32^, ^33^ were pooled in the analysis. The mean LOS range was 7–13.77 and 8–14.8 days in the prehabilitation and control groups, respectively.

The pooling WMD of any prehabilitation was −0.77 days in hospital stay, with low heterogeneity (I^2^ = 13%, *p* = 0.33) and statistical significance (95% CI = −1.46 to −0.09; Figure 4). In the multimodal/unimodal, ERAS/standard care, cancer type, and study design subgroups (Figure 4A–D), only patients with EC or gastroesophageal junctional cancer (WMD = −0.83, 95% CI = −1.54 to −0.12; Figure 4C) and non‐RCT studies (WMD = −1.02, 95% CI = −2.01 to −0.02; Figure 4D) provided statistically significant evidence favoring any prehabilitation decreasing LOS.

#### 3.5.3 Hospital readmission

**FIGURE 5:**
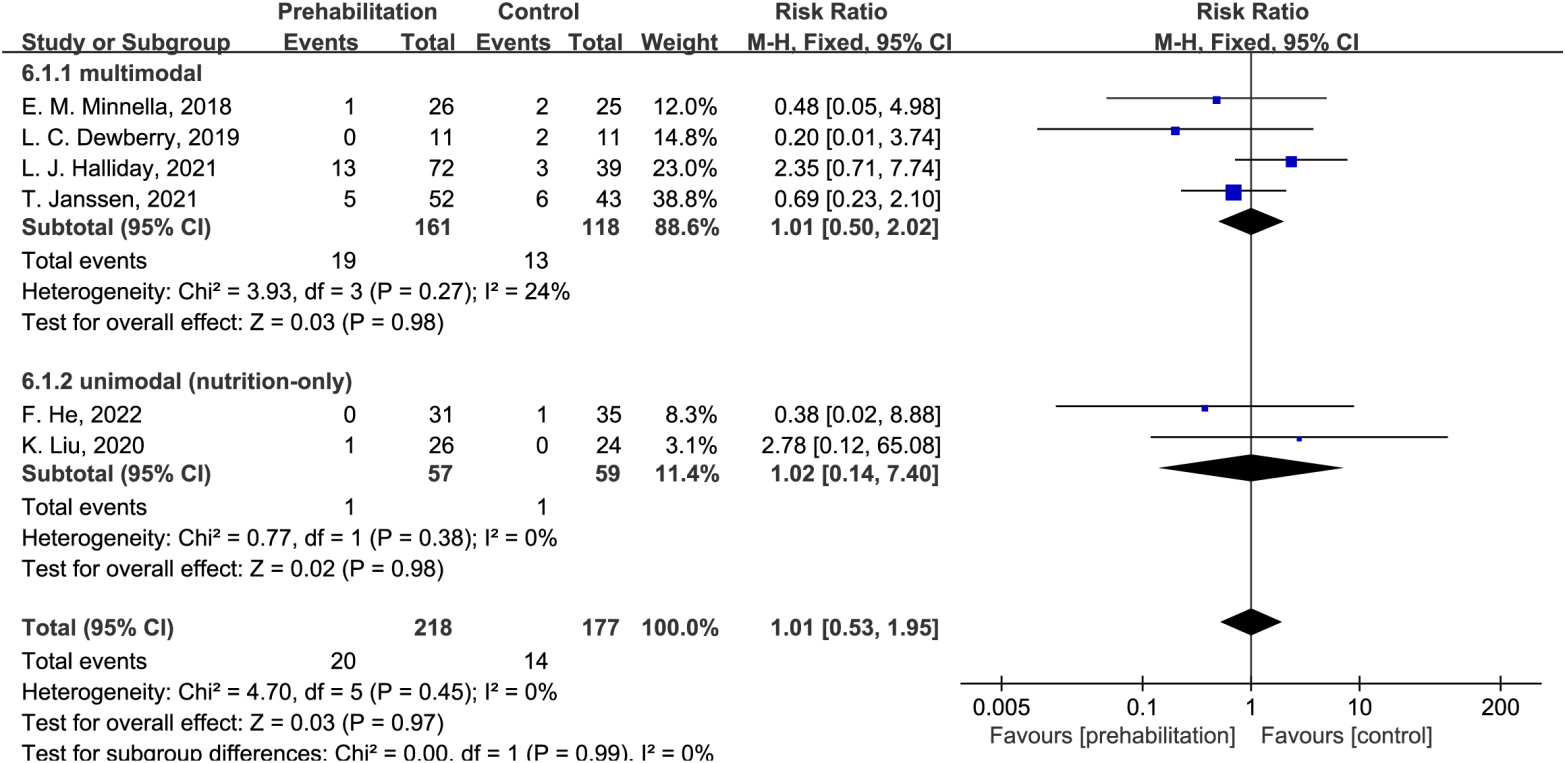
Effect of multimodal and unimodal (only nutrition) prehabilitation on hospital readmission in patients with esophagogastric cancer after esophagectomy and gastrectomy. CI, confidence interval.

The hospital readmission rate was reported in six studies^26^, ^27^, ^29^, ^31^, ^32^, ^33^ (Table 3 and Figure 5). However, two studies^27^, ^32^ did not provide a clear timescale, whereas others reported the rate within 30^26^, ^29^, ^31^, ^33^ or 90 days^31^ after discharge. In these six studies, the total readmission rate (within 30 days or without explicit time) was 9.2% (20/218) and 7.9% (14/177) in the prehabilitation and control groups, respectively. Then, hospital readmission was stratified by multimodal or unimodal prehabilitation. The overall pooled outcomes and subgroup analysis results suggested statistical insignificance for the effect of any prehabilitation on hospital readmission (Figure 5).

#### 3.5.4 Mortality

Mortality within 30 days^31^, ^32^ or 90 days^26^, ^31^, ^32^ after discharge or in the hospital^27^ was reported in four studies.^26^, ^27^, ^31^, ^32^ Owing to heterogeneity (different cutoff points for time) and low incidence (Table 3), a meta‐analysis of the effect of any prehabilitation on mortality could not be conducted. However, none of these studies conducted follow‐up and measured long‐term mortality.

## 4 DISCUSSION

Owing to their distinct characteristics, the pooled outcomes were stratified based on unimodal (nutrition‐only) and multimodal prehabilitation. Prehabilitation, where nutritional intervention was needed but the number of preoperative interventions was not restricted, could significantly decrease the risk of any postoperative complication, a primary complication of interest, by 23% after esophagectomy and gastrectomy. When stratified, multimodal prehabilitation generated a similar result; however, the results of unimodal prehabilitation were no longer statistically significant. Presumably, multimodal prehabilitation is superior to unimodal prehabilitation in terms of decreasing any postoperative complication. After esophagectomy and gastrectomy, postoperative pulmonary complications such as atelectasis, chylothorax, and pneumonia; severe complications (CDC grade 3 or higher); and anastomotic leakage were prevalent in patients with cancer, imposing adverse effects on the short‐ and long‐term outcomes, including morbidity and mortality; preventing such complications may improve overall survival.^3^, ^4^, ^35^ Therefore, we also analyzed these three types of postoperative complications. The outcomes indicated that prehabilitation decreases the risk of postoperative severe and pulmonary complications but not that of anastomotic leakage. Among them, multimodal prehabilitation may be superior to unimodal prehabilitation in decreasing severe and pulmonary complications; however, this finding should be further confirmed because only two RCTs^29^, ^32^ (*n* = 116) in the unimodal prehabilitation subgroup reported outcomes of a part of these complications. In this meta‐analysis, we discovered the benefits of prehabilitation that included nutritional interventions to decrease the incidence of any postoperative complication, including some common or severe complications, in patients with esophagogastric cancer. Among them, multimodal prehabilitation may confer more advantages than unimodal prehabilitation. A decrease in postoperative complications positively affects the quality of life, hospital days and costs, and overall survival of patients.^36^

In all clinical settings, the estimated effect of prehabilitation on LOS was small (mostly decreased by <1 day). Although LOS decreased, the extent of such a reduction is not extremely meaningful in clinical settings. Furthermore, such an outcome appears to contradict the significant decrease in any postoperative complication, which markedly prolongs LOS.^37^ We hypothesized the following reasons for this: (a) an enhanced recovery protocol program 2.0 planned the discharge day at 6 for patients after esophagectomy without or with minor complications (the shortest LOS to the best of our knowledge; in practice, the median discharge day was seven in this study).^6^ Because most of the included studies (3/5) followed the ERAS protocol in both groups, the mean LOS in the intervention and control groups was already remarkably similar to this idealized limit (6 days). Using any other intervention to further decrease LOS will be challenging. (b) Most of the included studies (4/5) expressed LOS using median and interquartile range; however, they were converted to mean and SD for statistical analysis. Some of these raw data may be skewed, and such conversions can distort the true effect. In the present study, we could only reveal that prehabilitation exerts a limited effect on decreasing the LOS of patients with esophagogastric cancer who had undergone surgery.

Preoperative nutritional support is a vital component of prehabilitation. In the included studies, ONS was predominantly administered to provide preoperative nutritional support because it is more convenient, less invasive, and has fewer complications associated with tube placement compared with EN or parenteral nutrition in patients without GI obstruction. However, special and repeated incentives are warranted because compliance with ONS intake is frequently limited.^38^ In patients with GI obstruction, either EN or parenteral nutrition can accelerate postoperative recovery.^39^ Because no specific evidence‐based recommendations are available, the target settings for energy and protein intake were markedly different in the included studies. We did not include studies that examined preoperative immunonutrition (e.g., arginine, ω‐3 fatty acids, nucleotides, and glutamine) because these agents are primarily utilized to improve host immune status and inflammatory responses^40^; this is inconsistent with the objective of our study. The implementation of preoperative nutritional interventions is based on three primary considerations: (a) to improve the nutritional status of the body in advance (focus on prevention) to optimally cope with the high catabolic state during and after surgery, inadequate nutrition intake resulting from postoperative fasting, and body depletion owing to possible postoperative complications, and not necessarily to replace nutritional deficits.^13^, ^41^ Nutritional interventions conducted some amount of time in advance, rather than waiting until the development of malnutrition, that is, a forward movement of the intervention gateway, is considered an excellent protocol in clinical settings.^42^ A superiority RCT to compare prehabilitation (nutrition and exercise) with rehabilitation (immediately after surgery) in patients undergoing colorectal resection for cancer revealed that prehabilitation can significantly improve the functional exercise capacity both preoperatively and postoperatively compared with rehabilitation.^43^ (b) The use of postoperative EN preparations often increases the incidence of nutrition‐associated complications, including vomiting, abdominal distention, and ventilator‐associated pneumonia.^44^ We hypothesize that preoperative ONS or EN facilitates a preadaptation process in the digestive tract to decrease the incidence of postoperative nutrition‐associated adverse effects and provides a faster transition from parenteral nutrition to EN, as well as the adequate use of established EN preparations after surgery, accelerating body recovery^45^ and allowing more nutrition intake, thereby establishing a positive cycle. (c) Preoperative exercise alone without nutritional supplementation may limit exercise intensity and duration, making it less effective. A study revealed that patients who did not achieve their preoperative exercise goals tended to exhibit an increased need for nutritional interventions at baseline.^46^

Although nutrition is essential, exercise can also play a vital role in promoting protein synthesis by muscles.^41^ The rational arrangement of exercise and nutritional supplementation preoperatively can increase the benefits of both measures for patients; for example, ingesting carbohydrates 3–4 h before exercise can increase liver and muscle glycogen reserves and improve the performance of endurance exercise.^47^ Hypertrophy in skeletal muscle in response to resistance exercise requires the early ingestion of an oral protein supplement after resistance training.^48^ In the currently included studies, nutrition and exercise were two independent interventions, and further exploring how they can be combined and interspersed more rationally and efficiently is worthwhile.

In this meta‐analysis, there were differences in the duration of prehabilitation interventions in the included studies, with a range of 1 to 10–18 weeks; this may have affected the interpretation of the results. In previous meta‐analyses of unimodal or multimodal prehabilitation for abdominal cancer surgery, similar variations in the duration of prehabilitation interventions have been observed, with a range of 2–14 weeks.^49^, ^50^ Subgroup analyses suggested that prehabilitation for more than 3 weeks decreased the overall complication rate compared with a shorter intervention time.^49^ However, the risk of cancer progression associated with a longer preoperative waiting time should be carefully considered. Because only three studies^27^, ^29^, ^31^ in our meta‐analysis revealed the actual duration of prehabilitation interventions, subgroup analysis was not performed based on intervention duration.

Only one study^27^ in this meta‐analysis evaluated the effect of prehabilitation on the changes in the functional capacity of patients and confirmed the improved functional capacity of the patients in the prehabilitation group both before and after surgery (absolute change in 6‐min walk distance, a primary indicator in the 6‐min walk test to determine the functional capacity^51^). If an intervention to preserve functional capacity is not rapidly implemented in catabolic patients, functional deterioration will occur.^52^ The improvement in the physical function owing to prehabilitation can improve compliance and allow patients with esophagogastric cancer to better complete their planned treatment.^27^

To the best of our knowledge, this is the first systematic review and meta‐analysis to investigate prehabilitation, thereby emphasizing the effectiveness of nutrition and multimodality, in patients with UGI cancer undergoing surgery. Minnella et al.^27^ were the first to conduct a pragmatic RCT of a structured preoperative conditioning intervention involving nutrition and exercise prehabilitation for patients with UGI cancer completed in 2017. One meta‐analysis on prehabilitation for colorectal surgery revealed shortened LOS (~2 days), earlier functional recovery postoperatively, and decreased postoperative complications.^19^ This is consistent with the conclusions drawn in this meta‐analysis, suggesting that prehabilitation positively affects the postoperative outcomes of total GI. A recent systematic review of prehabilitation for patients undergoing UGI surgery summarized the effect of different prehabilitation interventions (including single intervention or various combinations of nutritional, exercise, and psychosocial interventions) on patient biopsychosocial and service outcomes, finding that prehabilitation improved preoperative impairments and that multimodal prehabilitation appeared to achieve better outcomes.^53^ In the studies included in our meta‐analysis, nutrition‐based prehabilitation was also found to improve functional capacity^27^ or reduce weight loss^31^ in the preoperative period, but was not quantitatively synthesized due to the paucity of studies reporting preoperative outcomes and the variability of outcome metrics. Future studies could focus more on preoperative outcomes along with postoperative outcomes.

Our review has several limitations. First, the sample size was small (*n* = 661) and all studies did not report all the results of interest. Therefore, the results of subgroup analyses should be cautiously interpreted. Second, similar to other meta‐analyses, inevitable differences were observed in baseline data, concept definitions, and study designs across the included studies. Where possible, we assessed and decreased these differences using more stringent inclusion and exclusion criteria, statistical tests, and subgroup analyses. In one RCT,^32^ ONS was prescribed as a preoperative treatment regimen in the control group if the NRS2002 score was ≥3, potentially underestimating the effect of the intervention group. Because the high heterogeneity could not be decreased, we only qualitatively described postoperative mortality. Third, owing to the limited number of studies completed to date, we could not definitively judge which unimodal and multimodal prehabilitation was superior and what was the best intervention duration. Fourth, blinding patients and healthcare providers was challenging; therefore, a high performance bias was observed in RCTs and the effect of the interventions may have been overestimated.

Based on the findings of this review, we propose recommendations for future studies on prehabilitation. First, specific patient classifications, including the elderly or those with preoperative malnutrition or sarcopenia, should be further investigated to identify who benefits the most. Second, multimodal prehabilitation may decrease patient compliance owing to more cumbersome interventions in the multimodal setting; therefore, the advantages of multimodal over unimodal prehabilitation should be explored by considering patient compliance and the requirement of appropriate interventions such as the sequential combination of exercise and nutrition.^47^, ^48^ Furthermore, better monitoring and reporting of patient adherence are essential.^54^ Third, including patient‐oriented results such as nutrition‐associated complications and functional capacity, a major determinant of surgical prognosis,^55^ is vital. Furthermore, exploring the changes in body components is encouraged. Fourth, uniform definitions should be adopted for reporting complications, including the Esophagectomy Complications Consensus Group definition.^56^, ^57^ Fifth, prehabilitation is a relatively new approach in UGI surgery. Furthermore, results have not been published for 13 prehabilitation research protocols associated with esophagogastric cancer surgery (Figure 1). Therefore, an update of this meta‐analysis is expected.

## 5 CONCLUSIONS

Nutrition‐based prehabilitation, with nutrition as a vital component, can significantly decrease any postoperative complication, including severe and pulmonary complications, in patients with esophagogastric cancer undergoing surgery. Furthermore, multimodal prehabilitation may be more advantageous than unimodal prehabilitation in decreasing postoperative complications. However, prehabilitation to decrease LOS was not clinically significant because a significant reduction was not observed (0.77 days). Additional RCTs and an updated meta‐analysis are warranted in the future to acquire more convincing evidence.

## AUTHOR CONTRIBUTIONS

**Yi Shen:** Conceptualization (equal); formal analysis (equal); funding acquisition (equal); investigation (equal); methodology (equal); resources (equal); software (equal); validation (equal); writing – original draft (equal). **Zhuangzhuang Cong:** Conceptualization (equal); data curation (equal); funding acquisition (equal); methodology (equal); visualization (equal); writing – original draft (equal). **Qiyue Ge:** Conceptualization (equal); data curation (equal); formal analysis (equal); investigation (equal); methodology (equal). **Hairong Huang:** Conceptualization (equal); formal analysis (equal); investigation (equal); validation (equal). **Wei Wei:** Investigation (equal); methodology (equal); resources (equal); software (equal); validation (equal); visualization (equal). **Changyong Wang:** Investigation (equal); methodology (equal); resources (equal); software (equal); visualization (equal). **Zhisheng Jiang:** Data curation (equal); investigation (equal); methodology (equal); software (equal). **Yuheng Wu:** Conceptualization (equal); investigation (equal); methodology (equal); project administration (lead); supervision (equal); writing – review and editing (lead).

## FUNDING INFORMATION

This work was supported by the National Natural Science Foundation of China [grant number 82002454] and the Medical Scientific Research Project of Jiangsu Health Commission [grant number ZD2021011].

## CONFLICT OF INTEREST STATEMENT

Authors have no conflicts of interest to declare.

## REGISTRY NUMBER AND WEBSITE

This meta‐analysis was registered at https://www.crd.york.ac.uk/PROSPERO/ as CRD42022314766.

## Supporting information


Appendix S1.

Figure S1.

## Data Availability

All data generated or analyzed during this study are included in this published article and its supplementary information files.
