# Supplementary material for: Effect of nutrition‐based prehabilitation on the postoperative outcomes of patients with esophagogastric cancer undergoing surgery: A systematic review and meta‐analysis
Source: Cancer Med. 2024 Jul 13;13(14):e70023. doi: 10.1002/cam4.70023 (PMC11245637; doi:10.1002/cam4.70023)
Supplement: Supplementary file 1 — Appendix S1. [file CAM4-13-e70023-s002.docx]

**Supplementary** **Appendix.** Search Strategy

PubMed, Web of Science, Embase, and CINAHL

#1 esophag* OR esophag* neoplasm* OR esophag* cancer*

#2 stomach OR gastric OR gastric neoplasm* OR gastric cancer* OR stomach neoplasm* OR stomach cancer*

#3 ((Energy OR protein OR diet* OR food OR nutri*) adj3 counsel*) OR ((Energy OR protein OR diet* OR food OR nutri*) adj3 modif*) OR ((Energy OR protein OR diet* OR food OR nutri*) adj3 support*) OR ((Energy OR protein OR diet* OR food OR nutri*) adj3 optim*) OR ((Energy OR protein OR diet* OR food OR nutri*) adj3 supplement*) OR (pre-hab* OR prehab* OR pre hab*) OR ONS

#4 (#1 OR #2) AND #3

Cochrane Library

#1 MeSH descriptor: [Esophageal Neoplasms] explode all trees

#2 MeSH descriptor: [Stomach Neoplasms] explode all trees

#3 ((Energy OR protein OR diet* OR food OR nutri*) adj3 counsel*) OR ((Energy OR protein OR diet* OR food OR nutri*) adj3 modif*) OR ((Energy OR protein OR diet* OR food OR nutri*) adj3 support*) OR ((Energy OR protein OR diet* OR food OR nutri*) adj3 optim*) OR ((Energy OR protein OR diet* OR food OR nutri*) adj3 supplement*) OR (pre-hab* OR prehab* OR pre hab*) OR ONS

#4 (#1 OR #2) AND #3
