# Supplementary figures and images for: Effect of nutrition‐based prehabilitation on the postoperative outcomes of patients with esophagogastric cancer undergoing surgery: A systematic review and meta‐analysis
Source: Cancer Med. 2024 Jul 13;13(14):e70023. doi: 10.1002/cam4.70023 (PMC11245637; doi:10.1002/cam4.70023)

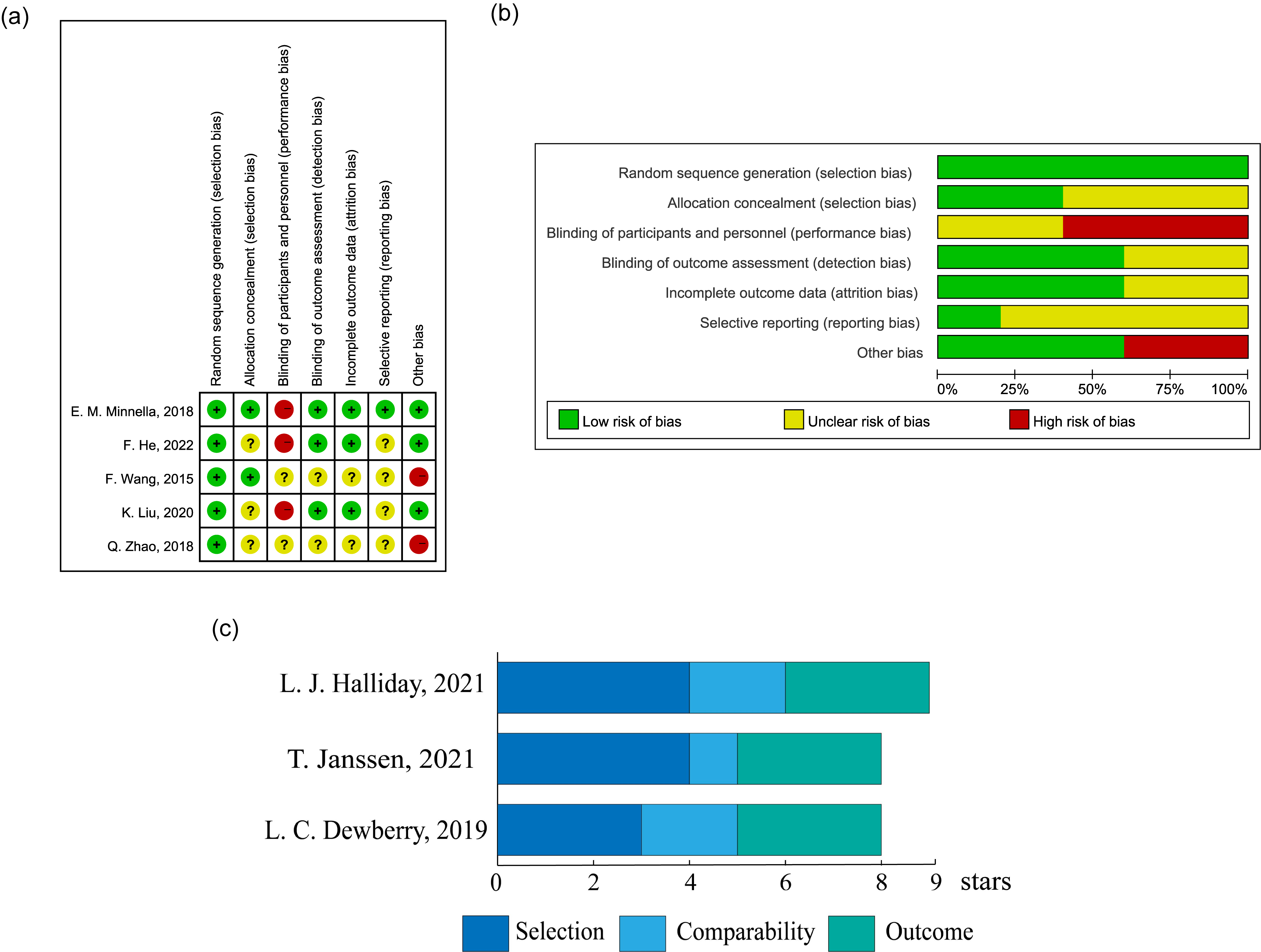

Supplement: Supplementary file 2 — Figure S1. [file CAM4-13-e70023-s001.tif]
